# Comparative Study of the Development of Executive Functions in Children: Transition from the First Cycle to the Second Cycle of Early Childhood Education

**DOI:** 10.3390/brainsci14121273

**Published:** 2024-12-18

**Authors:** Esperanza Bausela Herreras

**Affiliations:** Department of Health Sciences, Public University of Navarra, 31006 Pamplona, Spain; esperanza.bausela@unavarra.es

**Keywords:** BRIEF-P^TM^, development, early childhood education, executive functions, parents, teachers

## Abstract

Antecedents: Executive functions (EFs) are the basis for establishing a goal and working towards that goal by coordinating thoughts and actions. EFs are fundamental to several aspects of daily life, specifically for academic performance. Aim: To analyse and compare the development of EFs in the transition period between the first and second cycles of early childhood education. Methodology: Non-experimental methodology, ex post facto design, descriptive, cross-sectional evolution study. Participants: Preschoolers of different educational levels (first and second cycle of infant education). In this study, the participants were evaluated by different informants: 54.42% by parents and 45.58% by teachers. In relation to gender, 52.65% were male and 47.35% were female. In relation to age, 37.54% had a range of 2–3 years and 62.46% had a range of 4–5 years. Measurement: The instrumental development of EFs was evaluated using BRIEF-P by key informants. Results: Preschoolers in the first cycle showed significantly higher scores than preschoolers in the second cycle in BRIEP-P. Conclusions: The development of EFs is key in these first key moments, having a special impact on later development and academic performance. It is necessary to work on EFs from the first cycle of early childhood education, considering the evolutionary development of EFs.

## 1. Introduction

Garon, Bryson, and Smith [1] consider that brain development is a continuous process of construction, organisation, and re-structuring neural connections in response to environmental inputs, thus leading to an increase in the complexity of mental structures. These changes can be associated with and linked to the development of EFs [2].

### 1.1. Dimensions and Development of EFs in Early Childhood

Executive functions (EFs) are the basis of the ability to set a goal and then work towards that goal while coordinating thoughts and actions [3]. EFs are, therefore, fundamental to various aspects of daily life, such as mental and physical health, academic success, and success in daily living, along with social and psychological development [4,5]. They include diverse dimensions, such as cognitive flexibility, inhibitory control, and working memory [6,7,8], which are essential for functioning in human society.

EFs include processes that involve intentionality in the control of other processes, such as impulse control, attention, thought, and behaviour [2,9].

For Lezak [10], EFs are essential mental capacities for carrying out effective, creative, and socially adapted behaviour. Sohlberg and Mateer [11] consider that EFs cover a series of cognitive processes, among which are anticipation, selection of objectives, planning, behaviour selection, self-regulation, self-control, and the use of feedback. Tirapu [12], for example, focuses on predicting the consequences that can lead us to anticipated solutions. Funahashi [13] postulates that they are the result of the coordination of the processes that are necessary to meet a particular objective in a flexible way. Zelazo [14] focuses on the self-regulation skills involved in achieving a goal, which are modulated by thinking and emotion, while basically differentiating between executive and motivational executive processes or “hot EFs” and purely cognitive aspects or “cool EFs”.

The construct of EFs does not represent a unitary concept. Regarding the dimensions that constitute EFs in the early stages of development, study results are contradictory and inconclusive.

During the preschool stage, the dimensions of executive functions have been studied primarily through factor analyses (see [15]). These techniques allow for the exploration of the degree of independence or interdependence among the different components of the construct [16,17,18].

According to Monette, Bigras, and Lafrenière [16], results are more consistent at age 3 compared to ages 4 to 5 [19,20,21]. A three-factor solution suggests greater differentiation of the dimensions and appears more suitable for older children, between 8 and 13 years, or even up to around 15 years [22,23]. On the other hand, some studies indicate that the structure of EFs in children aged 3 to 5 years or 5 to 13 years may be based on two factors [23,24]. Other research supports a single-factor model for 5-year-old children [16], as well as for age ranges of 2.5 to 6 years [25] and 7 to 9 years [26]. Brydges, Fox, Reid, and Anderson [26] reviewed the literature and suggest that a single-factor model is more appropriate for children under 9 years of age.

There is no consensus on the number of factors that make up executive functions, as this construct varies throughout development. Various authors, such as [15], have used factor analyses to identify the underlying components, as detailed in the review conducted. The most recognised model is Miyake’s [2], which identified three key factors: updating, inhibition, and shifting. However, in the study by Spiegel, Lonigan, and Phillips [27], a discrepancy in the structure of the BRIEF-P was observed, with the best solutions proposed being a four-factor model and a bifactorial model.

There is growing evidence indicating that EFs are essential both for academic success and for the development of cognitive skills [28,29,30,31,32,33,34]. Significant improvements in EF task performance have been observed between the ages of 3 and 6 years [20,35].

The structural, functional, and neurological changes occurring in the prefrontal cortex are accompanied by changes in executive functions [15,36,37,38], as well as by the differentiation of executive functions throughout the lifespan, with each dimension following its own trajectory. Table 1 provides an outline of the development of EFs from 0 to 6 years, according to Zelazo and Carlson [38].

The development of EFs is a crucial aspect of cognitive growth in children, especially during the early years of life [38]. These skills, which include working memory, inhibitory control, and cognitive flexibility, play a fundamental role in children’s ability to plan, solve problems, and regulate their behaviour [15]. In the context of early childhood education, analysing the development of these functions can provide valuable insights into how children progress through different educational stages.

### 1.2. Evolution of Cognitive and Social Development Across Early Childhood Education Cycles

In terms of cognitive and psychological development, students in the first cycle (ages 0–3) are primarily engaged in sensory exploration and motor activities [39]. Their learning is driven by direct experiences and free play, with a focus on developing basic skills such as fine and gross motor coordination, imitation, and emerging language abilities [40,41]. At this stage, children are beginning to develop self-regulation and autonomy but still depend heavily on adults for guidance and decision-making. In contrast, during the second cycle (ages 3–6), students begin to acquire more advanced cognitive skills. They develop working memory, sustained attention, and problem-solving abilities [42,43]. There is also a notable expansion in language use, allowing them to follow complex instructions and express their thoughts and emotions more clearly. Additionally, their capacity for emotional and social regulation improves, and they start engaging in more complex and cooperative social interactions [44].

The educational environment also evolves significantly between these two cycles (see [45]. In the first cycle, the learning environment is highly stimulating and designed for safety, with an emphasis on exploration and free play. Educators act as guides, facilitating learning through interactive play. By the second cycle, the environment becomes more structured, incorporating daily routines and organised activities, including guided play and group tasks. The focus shifts toward preparing children for primary school with an introduction to pre-academic skills such as letter and number recognition and the ability to follow instructions and work in groups.

In the study developed by [46], the unique features of early childhood education in Spain are explored, emphasising its internationalised educational framework, comprehensive educational objectives, and well-developed welfare system. This framework includes a focus on sensory–motor development, early socialisation, and basic language skills as key goals for the first cycle of education. The emphasis on these developmental milestones aligns with Spain’s approach to fostering children’s overall growth, including their foreign language proficiency and digital literacy. This comprehensive approach not only highlights Spain’s educational strengths but also provides valuable insights for the development of early childhood education systems in other countries, particularly in terms of integrating socio-emotional and cognitive development with a global perspective.

The results of the systematic review by [47] emphasise the need for an interdisciplinary, play-based developmental assessment tool. This tool would support teachers in guiding developmentally appropriate teaching and learning processes, as well as contributing to research and psychopedagogy fields. Evaluation during this period is more observational, focusing on progress in these fundamental areas. In the second cycle, learning goals become more formal, aiming at preparing children for primary education. This includes a focus on initial academic skills, understanding basic concepts and working on structured tasks. Assessment methods become more formal, although still adapted to the child’s age, to evaluate cognitive, linguistic and social development [48].

Sandstrom [49] explores the crucial role of the curriculum as a strategic tool to enhance the quality of early childhood education and clearly and concisely describes the structure of the early childhood education stage in Spain, highlighting its main characteristics. The first and second cycles of early childhood education represent key phases in this developmental process. While the first cycle focuses on initial exploration and the acquisition of basic skills, the second cycle is oriented towards a greater consolidation and refinement of these abilities. The transition between these two cycles can be a critical period for the development of EFs (see [50]), marking a significant change in how children manage their cognitive and emotional skills.

Social interaction and behaviour notably change between the two cycles [51,52]. In the first cycle, social interactions are simpler, typically involving imitation and parallel play, with early development in understanding social norms and cooperative play. By the second cycle, children engage in more cooperative and group activities, showing improved understanding and adherence to social rules, as well as enhanced abilities to share and collaborate with peers.

These differences underscore a significant evolution in children’s development as they advance from the first to the second cycle of early childhood education, reflecting their cognitive, social, and emotional growth.

Given the potential impact that this transition may have on the development of EFs [53], it is essential to investigate how the level of development of these skills varies between children in the first and second stages of early childhood education. Additionally, identifying the differences in development during the transition period between the two cycles is important for better understanding the needs and appropriate supports for children at this crucial stage of their development [54,55].

### 1.3. Aim and Research Hypotheses

Supported by Blair’s study [56] on executive functions, which highlights their rapid development in early childhood and their importance for academic success, we have based our hypotheses and objectives on his work.

We set ourselves the objective of knowing, analysing, and comparing the level of development of the EFs of infants in the first cycle of infant education versus the second cycle, the age of intercycle transition (first versus second).

The transition from the first stage to the second cycle in early childhood education marks several significant changes in the developmental, educational, and behavioural aspects of students. These differences are crucial for understanding how children evolve as they move through these formative years.

How does the level of executive function development in children in the first stage of early childhood education compare to that of children in the second stage, and what differences are observed in the development of these functions during the transition period between the two stages?

It is expected that children in the second cycle of early childhood education (4–5 years) will show a higher level of development in executive functions, specifically in areas such as attention, working memory, and inhibition, compared to children in the first cycle (2–3 years), due to neurocognitive maturation and the increasing cognitive demands during this transition stage in child development.

## 2. Materials and Methods

We set out to create a non-experimental, ex post facto design, descriptive, cross-sectional development study.

### 2.1. Design

The study procedure is based on a non-experimental methodology, with an ex post facto and descriptive design, in which the evolution of executive functions in children is analysed during the transition between the first and second cycles of early childhood education. A cross-sectional study was conducted, in which participants, coming from different educational levels (the first and second cycles of early childhood education), were evaluated by key informants, specifically parents and teachers.

The instrument used for evaluating executive functions was the BRIEF-P (adapted from [57]), which provides information on self-regulation and other cognitive functions of children in an educational context. The sample includes children aged between 2 and 5 years, with a balanced distribution of gender and age, ensuring representation of both stages of child development. The study compares the results obtained from children in the first cycle (2–3 years) with those in the second cycle (4–5 years), allowing for the identification of significant differences in the development of executive functions between the two groups.

This approach provides a clear view of the developmental differences in children’s executive functions, facilitating the understanding of how these functions develop as children progress through early childhood education.

### 2.2. Participants

The typing sample is constituted by 1042 boys and 937 girls who are participants in the process of adaptation and validation of BRIEF-P (adapted from [57]). The inclusion criteria were to acceptance participation in the study, age range between 2 and 6 years, and no signs or indications of any type of neurodevelopmental disorder and/or disability.

In this study, the participants have been evaluated by different informants: 54.42% by parents and 45.58% by teachers. In relation to gender, 52.65% were male and 47.35% were female. In relation to age, 37.54% were in the range of 2–3 years and 62.46% were in the range of 4–5 years.

Students were grouped by education cycle and age. Table 2 shows the distribution of the participants according to educational cycle.

### 2.3. Measurement

Executive function assessment with BRIEF-P is an instrument that was recently validated in Spain by Bausela and Luque [57] with the aim of evaluating its development through the observation of key informants (teachers or other habitual caregivers of the child) (hetero research, self-investigation).

The BRIEF-P provides scores on various indices (global index of executive function, inhibitory self-control index, flexibility index, emergent metacognition index) and scales related to EFs (inhibition, flexibility, emotional control, working memory, planning and organisation).

BRIEF-P was completed by parents, legal guardians, and teachers of children with ages from 2 years to 5 years and 11 months who have had knowledge of the child for a minimum period of 6 months. The study was carried out using individual and collective applications.

The psychometric properties can be consulted in Bausela and Luque [58], where the relevant aspects related to reliability, validity, and other essential characteristics of the instrument used are detailed. For more information, see the Supplementary Materials.

### 2.4. Procedure

The procedure for applying the BRIEF-P by parents and teachers involves four key stages to ensure the validity and proper implementation of the test. First, collaborators must familiarise themselves with the norms, thoroughly reading them to understand the guidelines, securely storing materials, and explaining the evaluation objectives and data handling to informants. Second, participants are randomly selected from a normal sample without prior diagnoses, as are informants, including parents or teachers familiar with the child’s behaviour. Third, differentiated booklets are used—one for parents and another for teachers/caregivers—and informants must complete their respective booklets while maintaining confidentiality and returning them appropriately. Finally, data are entered into the scoring program at www.teacorrige.com, generating a graphical profile, intended as a general guide rather than a clinical diagnosis.

### 2.5. Analysis of Data

The data were submitted to descriptive and inferential analyses (bivariate and multivariate). Student’s t-test was used to calculate the difference in the scales and clinical indices of BRIEF-P between the two age groups by age and educational cycle.

## 3. Results

The initial development includes descriptive statistical analyses, followed by inferential analyses.

Table 3 and Table 4 show the distribution of the sample of participants evaluated according to the informants (parents versus teachers) in the two age groups (three years versus four years) and educational cycle (first cycle versus second cycle of early childhood education) that were evaluated.

Table 5 and Table 6 show the results of the Levene test of equality of variances prior to Student’s *t*-test for the equality of means (or medians) of independent samples of the two informants (parents-teachers).

The difference between first- versus second-cycle students was different depending on the informant:

(i) When parents are informants, there are no statistically significant differences in planning and organisation, inhibitory self-control, and emergent metacognition. In all clinical scales and indices, except in planning and organisation, scores are higher in first-cycle participants compared to second-cycle participants (see Table 5).
  There are statistically significant differences in three clinical scales: flexibility (*t* [1075] = 3.94, *p* = 0.000, *d* = 0.014), emotional control (*t* [1075] = 1.373, *p* = 0.17, *d* = 0.002), and working memory (*t* [1075] = 1.915, *p* = 0.056, *d* = 0.003); and in two indices: flexibility (*t* [1075] = 3.049, *p* = 0.002, *d* = 0.009) and global executive performance (*t* [1075] = 1.714, *p* = 0.087, *d* = 0.003).
(ii) When teachers are informants, there are statistically significant differences (see Table 6).
  These differences were found in all clinical scales and indices. In all clinical scales and indices, the scores are higher in participants in the first cycle compared to those in the second cycle. Statistically significant differences are obtained in:
  (a) Three clinical scales: inhibition (*t* [900] = 3.127, *p* = 0.002, *d* = 0.002), flexibility (*t* [900] =5.565, *p* = 0.000, *d* = 0.000), emotional control (*t* [900] = 3.668, *p* = 0.000, *d* = 0.000), working memory (*t* [900] =4.179, *p* = 0.000, *d* = 0.000), and planning and organisation (*t* [900] = 4.381, *p* = 0.000, *d* = 0.000).
  (b) All clinical indices: inhibitory self-control (*t* [900] = 3.622, *p* = 0.000, *d* = 0.000), flexibility (*t* [900] = 5.249, *p* = 0.000, *d* = 0.000), emergent metacognition (*t* [900] = 4.377, *p* = 0.000, *d* = 0.000), and global executive function (*t* [900] = 5.08, *p* = 0.000, *d* = 0.000).

Although the differences in means are significant in both groups, the effect sizes reported in parent evaluations are generally smaller, indicating that the observed differences, while statistically significant, are not substantial in practical terms. In contrast, teacher ratings show larger effect sizes, suggesting that the differences in executive functions observed by teachers are more pronounced and may have greater practical relevance. Thus, larger differences between the two educational stages are observed in the clinical working memory scale, the emergent metacognition index, and the global executive functioning index. When parents are the informants, the greatest differences reported between the two educational stages are found in the clinical flexibility index, flexibility index, and global executive functioning index.

These results suggest that, although there may be some agreement between parents and teachers regarding children’s executive functions, the differences in effect sizes imply that teachers are observing more pronounced issues or skills related to executive functioning than parents. This could reflect differences in settings (home vs. school) or the nature of tasks in each context.

## 4. Discussion

The objective of the present study was to analyse and compare the development of EFs among schoolchildren who are in the transition period between the first and second cycles of early childhood education, in order to identify the executive dimensions that are developed in this educational period, which will mark subsequent development and academic performance.

The choice of two groups of schoolchildren with very different ages allowed us to determine whether, in these first periods of development, EFs form a segment or set, or whether, on the contrary, the dimensions that make up this construct are already differentiated according to different rhythms in their trajectory.

When the comparison is made using educational cycles, the differences increase in the number of indices and clinical scales within which there are statistically significant differences, particularly, in the case of parents, having significant differences in inhibition, flexibility (index and scale) emotional control, working memory, and global executive function. In the case of teachers, there are still statistically significant differences between schoolchildren in both cycles at all scales and clinical indices. It can be affirmed that when the comparison is made by educational level, a greater number of dimensions are obtained in which there are statistically significant differences among schoolchildren to substantiate design intervention programs aimed at the development of EFs in early childhood education.

The results obtained are in line with the results obtained by [19], who allude to the fact that at 3 years of age, more complex executive skills are developed (conflict resolution or active manipulation of information in working memory). At this same age, some 3-year-olds are flexible with respect to their attention in response to the demands of the situation and change, for example, their responses following clear verbal instructions. The foundations of planning and organisational skills have been evidenced in children aged 3 and 4 years [59].

In relation to inhibitory control, the results obtained take on the same direction as those obtained by Pérez, Carboni, and Capila [12], who conclude that in tasks requiring set change (e.g., Wisconsin Card Sorting Test) 3-year-olds are unable to inhibit the mental set in which they are currently engaged and redirect their attentional focus to the new set.

We can consider that the stage between 3 years and 5 years of age is the period in which there is improvement of the processes of memory and inhibitory control that have arisen in early childhood (0 years of age). These basic skills will be fully developed in the school year.

Regarding cognitive flexibility, the results also confirm what Diamond [4] pointed out earlier, which is that it began to develop later in the period of 2 years and 10 weeks to 3 years of age, showing a significant increase during the school years.

Concerning self-regulation abilities, the data obtained are consistent with those that indicate that from 3 years and 6 months of age, a new stage is reached in terms of the inhibitory properties of adult speech, which is intended to be the child’s own voice as the factor that achieves some voluntary control. This is achieved in children between the ages of 3 and 4 years as, during this period, the impulsive function of children’s speech continues to predominate, although it slowly gives way to a semantic control that in later stages will really be determinant.

Functions of the central executive (inhibitory control, working memory, and cognitive flexibility) are especially important in this stage of early childhood education, conditioning subsequent development and access at the primary education stage. At the end of the first cycle of early childhood education, it is expected that the child will be able to initiate skills in logical/mathematical intelligence and reading/writing, among others.

In relation to the informant, we can state in general terms that parents and teachers are reliable sources for assessing development of EFs in early childhood education. However, in agreement with other authors, there are differences and similarities in their perception of the development of EFs [60,61]. These results may indicate that, when the teachers are the informants, they are more sensitive to development compared to the parents. On the other hand, children 3 years of age and in the first cycles present more difficulties with EFs, but these difficulties are reduced in the following year as a result of their own neurological maturation and their own educational intervention, which is developed in infantile education.

The obtained data indicate that when the informants are the teachers, statistically significant differences are obtained on all scales and clinical indices of BRIEF-P, both when the comparison is made by educational cycles as well as by chronological age.

When the informants are the parents, statistically significant differences are obtained on a smaller number of scales and clinical indices of BRIEF-P. Specifically, when the comparison is made by age and the informants are the parents, we obtain differences in scale and clinical index flexibility. When the comparison is made equally by age by teachers, statistically significant differences are obtained in all scales and clinical indices.

Thus, we researched how to analyse the degree of “sensitivity” that parents and teachers have toward this development, and also how to know the variable that has the greater predictive power of the performance of the EFs. The absence of statistically significant differences in the development of EFs in the two age groups (first cycle versus second cycle of early childhood education) was hypothesised in this study, since the study dimensions that configure the construct of EFs are interrelated with the first periods of development. Furthermore, it is not easy for observers (parents and teachers) to discern between the two groups of participants when comparing two groups of subjects with very similar ages, with a very small difference between the two educational cycles.

The development of EFs is analysed differently by informants (parents versus teachers), with significant differences between respondents. Thus, when the informants are teachers, statistically significant differences are obtained between the two age groups that are being analysed, whereas when the informants are the parents, there are no statistically significant differences in different clinical scales (inhibition and planning and organisation) and in clinical indices (inhibitory self-control and emerging metacognition). The differences found among the observers are in line with the results obtained by other researchers [60,61,62,63].

The obtained results allow us to acknowledge the existence of statistically significant differences according to the two age groups that were analysed, when evaluated by both parents and teachers. These data confirm that the different executive dimensions are supported by general neural circuits that mature and change throughout the life cycle, making it possible to analyse and make comparisons from the first periods of the life cycle. Our data, as well as those provided by other studies [1,64,65], suggest that as the development of typical children progresses, the children are maturing with respect to their executive competencies. This means that those children are more able to inhibit automatic responses that positively affect their attentional capacity, are more flexible, have greater emotional control, and are more capable of storing and managing information [66] in the field of mathematics [67] and in other fields of study. This typical developmental milestone will allow us to understand the difficulties that some children have in these competences, for example, children with attention deficit hyperactivity disorder [68,69], children with low birth weight (Anderson, McNamara, Andridge, and Keim, 2015) [70], children with language disorders [71] or learning disabilities [72], or children with autism spectrum disorder [73,74].

We can conclude by affirming that BRIEF-P is an instrument that is sensitive to the development of EFs and it is the informant that interacts with the child, guaranteeing a plural and diverse view depending on the development contexts (home versus school) and ecological validity of the scores. These results are in line with results obtained by other researchers [75] and justify the creation of different scales according to age groups and educational cycle. It is observed that the different dimensions evaluated (emergent metacognition, inhibitory self-control, and flexibility) follow different trajectories, being in line with the approach proposed by several researchers in relation to EFs [4,28,76,77,78].

These results lead to a need to proffer intervention proposals that are sensitive to these differences in development during the first stage of the life cycle, avoiding consideration of the EFs as a segment or set, since, for this first stage, evidence of differences in their development has been verified among children of age groups in the first cycle versus second cycle of early childhood education [3,79]. These results are consistent with neuroimaging studies, which confirm that the maturation of neural connections occurs by following a process, from the interconnected local regions to the regions of distributed connectivity, which works as the basis of the same function, as reflected in the maturation of cognitive abilities [80].

In the case of cognitive flexibility, the data are in line with those data provided by [3], who indicate that flexibility emerges towards the end of the third year of life, thus being at the border between the two age groups that were analysed.

The study highlights that, while there are significant differences in executive functions according to reports from parents and teachers, the effect sizes indicate that these differences are much more substantial from the teachers’ perspective. However, both sources of assessment provide valuable information, and future research could explore why such discrepancies exist and whether interventions in either domain (home or school) could account for these differences.

These results also force us to include teachers and parents as evaluation agents and not to ignore their contributions. In accordance with this, the need to train and instruct parents and teachers in this construct is also estimated (for example, EFs and their relationship with basic instrumental skills, such as mathematical competence). The deep knowledge of the qualitative changes (and not limited to the quantitative aspects) of these higher-order cognitive processes are necessary to obtain a referent norm and to be able to understand all the differences and/or deviations that occur in typical development or disorders from the early stages of the life cycle (primary intervention) [81].

One of the strengths that we estimate from the present study is the sample that has been extracted from all of the national territory, which guarantees the representativeness and generalisation of the obtained results.

A potential limitation of this study is its cross-sectional design, which, although it provides a snapshot of the development of EFs at different stages of early childhood education, does not allow for the observation of longitudinal or dynamic changes over time in the same individuals. This approach may limit the ability to identify specific developmental patterns or analyse the impact of variables that evolve over time.

Nevertheless, the results obtained remain significant, as they provide a solid foundation for understanding the developmental differences between the first and second cycles and highlight the importance of fostering EFs from early childhood. Future studies could complement this work with longitudinal designs to corroborate and expand upon the conclusions presented here.

We are interested in opening new lines of research in collaboration with the Nesplora team, with the aim of analysing the concurrent validity of the BRIEF-P by comparing it with tests that assess executive functions using virtual reality technology. This collaboration will allow us to explore how virtual-reality-based assessments, which simulate dynamic and realistic environments, can complement or enrich traditional approaches to measuring executive functions. Through this comparative analysis, we aim to deepen the accuracy and applicability of assessment tools, as well as advance the development of innovative methods for studying cognitive performance in contexts closer to real-life situations [82,83].

Finally, in future lines of work, we will explore some of the factors that other authors [84,85] have investigated to explain the development of EFs. These factors include, among others, environmental influences, developmental trajectories, and individual and group differences, as well as the interactions between general cognitive abilities and specific experiences. This approach will allow us to expand our understanding of the processes underlying the development of EFs and its variability across different life stages and contexts.

## 5. Conclusions

In the present study, a crosscutting design has been proposed, which makes it impossible to analyse intraindividual differences in the development of EFs; it is therefore necessary to choose longitudinal designs [86] in order to analyse and compare intraindividual changes in the development of EFs.

## Figures and Tables

**Table 1:** Functional development of executive functions from 0 to 6 years (adapted from [38]).

Age	Cold EFs	Hot EFs
7–8 months	Early signs of working memory and inhibition	Able to distinguish between animate and inanimate objects
12 months	-	Focused attention
14 months	-	Social referencing
2 years	Improvements in inhibition and working memory	Understanding of pretend play situations
3 years	Improvements in inhibitory control and attention up to age 5	Enhancements in affective decision-making this year
4 years	Improvements in cognitive flexibility	Successfully completes “false belief” tasks
5 years	Gains in working memory and strategy development. Begins to plan and direct behaviour toward goals.	Aware that beliefs can be based on other beliefs
6 years	-	Theory of mind becomes as sophisticated as an adult’s

**Table 2:** Distribution of participants according to age and educational cycle (own elaboration).

Cycle	Year	Parents	Teachers	Total	%
1°	2–3 years	418	325	743	37.54%
2°	4–5 years	659	577	1236	62.46%
Subtotal		1077	902	1979	100%

Source: BRIEF-P (Spanish adaptation).

**Table 3:** Distribution of the sample according to age and educational cycle by informant (parents) (only elaborated).

Parents ^(a)^					
Clinical Scales/Indices		1° Cycle ECE		2° Cycle ECE	
		M	σ	M	σ
Clinical Scales	Inhibition	23.97	5.589	23.89	5.789
	Flexibility	13.96	3.363	13.17	3.05
	Emotional Control	14.67	3.538	14.36	3.661
	Working Memory	23.75	5.589	23.09	5.479
	Planning and Organisation	14.38	3.002	14.39	3.517
Indices	Inhibitory Autocontrol	38.64	8.417	38.25	8.673
	Flexibility	28.63	5.792	27.53	5.705
	Emergent Metacognition	38.13	8.126	37.48	8.578
	Global Executive Function	90.73	16.58	88.9	17.29

^(a)^ Parents: *n* = 1077. Source: BRIEF-P (Spanish adaptation). ECE: early childhood education.

**Table 4:** Distribution of the sample according to age group and educational cycle by informant (teachers) (only elaborated).

Teachers ^(a)^					
Clinical Scales/Indices		1° Cycle ECE		2° Cycle ECE	
		M	σ	M	σ
Clinical Scales	Inhibition	22.77	6.255	21.42	6.193
	Flexibility	13.46	3.354	12.26	2.967
	Emotional Control	13.74	3.804	12.8	3.651
	Working Memory	23.64	6.747	21.81	6.015
	Planning and Organisation	13.82	3.919	12.76	3.235
Indices	Inhibitory Autocontrol	36.51	9.183	34.22	9.085
	Flexibility	27.2	6.238	25.06	5.604
	Emergent Metacognition	37.46	10.357	34.57	8.987
	Global Executive Function	87.44	19.278	81.05	17.43

^(a)^ Teachers: *n* = 902. Source: BRIEF-P (Spanish adaptation). ECE: early childhood education.

**Table 5:** Independent sample test in relation to age (first cycle versus second educational cycle of early childhood education) (version for parents) (own elaboration).

Executive Function		Parents ^(a)^						
		Levene Test of Equality Variances		Student’s *t*-Test for the Equality of Means				
		F	Sig. (Bilateral)	*t*	gl	Sig. (Bilateral)	Difference in Means	*d*
Clinical Scales	Inhibition	1.258	0.262 n.s.	0.215	1075	0.83 n.s.	0.077	0.000
	Flexibility	4.49	0.034 *	3.941	1075	0.000 ***	0.782	0.014
	Emotional Control	0.614	0.433 n.s.	1.373	1075	0.17 **	0.31	0.002
	Working Memory	0.005	0.941 n.s.	1.915	1075	0.056 *	0.661	0.003
	Planning and Organisation	10.234	0.001 ***	–0.047	1075	0.962 n.s.	–0.01	0.000
Indices	Inhibitory Autocontrol	0.826	0.364 n.s	0.726	1075	0.468 n.s.	0.389	0.000
	Flexibility	0.047	0.828 n.s.	3.049	1075	0.002 **	1.094	0.009
	Emergent Metacognition	2.79	0.095 ***	1.239	1075	0.216 n.s.	0.651	0.001
	Global Executive Function	1.268	0.26 n.s.	1.714	1075	0.087 *	1.824	0.003

Source: BRIEF-P (Spanish adaptation). *** *p* < 0.01, ** *p* < 0.05, * *p* < 0.1, n.s. not significant. ^(a)^ Parents: 1° cycle early childhood education *n* = 418/2° cycle early childhood education *n* = 659.

**Table 6:** Independent sample test in relation to age (first cycle versus second educational cycle of early childhood education) (version for teachers) (own elaboration).

Executive Function		Teachers ^(a)^						
		Levene Test of Equality Variances		Student’s *t*-Test for the Equality of Means				
		F	Sig. (Bilateral)	*t*	gl	Sig. (Bilateral)	Difference in Means	*d*
Clinical Scales	Inhibition	0.115	0.734 n.s.	3.127	900	0.002 **	1.348	0.002
	Flexibility	12.309	0.000 ***	5.565	900	0.000 ***	1.201	0.000
	Emotional Control	2.626	0.105 *	3.668	900	0.000 ***	0.943	0.000
	Working Memory	5.759	0.017 **	4.179	900	0.000 ***	1.822	0.000
	Planning and Organisation	11.969	0.001 ***	4.381	900	0.000 ***	1.062	0.000
Indices	Inhibitory Autocontrol	0.393	0.531 n.s.	3.622	900	0.000 ***	2.291	0.000
	Flexibility	8.272	0.004 ***	5.294	900	0.000 ***	2.144	0.000
	Emergent Metacognition	6.856	0.009 ***	4.377	900	0.000 ***	2.885	0.000
	Global Executive Function	3.502	0.062 *	5.08	900	0.000 ***	6.383	0.000

Source: BRIEF-P (Spanish adaptation). *** *p* < 0.01, ** *p* < 0.05, * *p* < 0.1, n.s. not significant. ^(a)^ Teachers: 1° cycle early childhood education *n* = 325/2° cycle early childhood education *n* = 577.

## Data Availability

These data have been provided to complement the information presented in the text, while fully respecting the confidentiality and privacy established as co-authors of the BRIEF-P Spain adaptation, as well as the commitments signed with TEA Ediciones Publishing. These data have been managed with due ethical consideration and in accordance with the established agreements, ensuring the protection of sensitive information.

## Supplementary Materials

The following supporting information can be downloaded at: https://www.mdpi.com/article/10.3390/brainsci14121273/s1.
