# Supplementary material for: Comparative Study of the Development of Executive Functions in Children: Transition from the First Cycle to the Second Cycle of Early Childhood Education
Source: Brain Sci. 2024 Dec 18;14(12):1273. doi: 10.3390/brainsci14121273 (PMC11674397; doi:10.3390/brainsci14121273)
Supplement: Supplementary file 1 [file brainsci-14-01273-s001.zip › PROPIEDADES PSICOMÉTRICAS EN ESPAÑOL DE BRIEF-P_SPANISH.pdf]

Materiales Complementarios Psychometric Properties In Spanish Of BRIEF-P

Information extracted from:

Bausela-Herreras E, Luque-Cuenca T. Evaluación Conductual de la Función Ejecutiva-Versión Infantil (BRIEF-P, versión española): fiabilidad y validez. Acta de Investigación Psicológica. diciembre de 2017;7(3):2811-22.
DOI: 10.1016/j.aipprr.2017.11.002

"Seguidamente se presentan los resultados obtenidos del estudio de las propiedades psicométricas de BRIEF-P en su adaptación española:

I) En relación a la *fiabilidad* se consideró la *Consistencia interna*: Se ha calculado a través del coeficiente α de Cronbach, obteniéndose, los siguientes coeficientes: (i) padres y varones [α= .906] (ii) padres-mujeres [α =.908], (iii) profesores-varón [α =.901] y (iv) profesores-mujeres [α =.903]. En todas las submuestras estudiantes el coeficiente de fiabilidad obtenido es superior a .90, pudiendo afirmarse que es excelente. Seguidamente se presentan los coeficientes de fiabilidad en las diferentes escalas e índices clínicos del BRIEF-P en función del informante y del sexo.

Tabla S1. Estadísticas de fiabilidad en función de los informantes (padres versus profesores) y sexo.

Informante	Sexo	Alfa de Cronbach	Alfa de Cronbach basada en elementos estandarizados	Número de elementos
Padres	Varón	.906	.945	9
	Mujer	.908	.947	9
Profesores	Varón	.901	.942	9
	Mujer	.903	.944	9

Fuente: BRIEF-P (adaptación española).

b) *Consistencia temporal o estabilidad test – retest:* Índice Global de Función Ejecutiva los coeficientes fueron [α=0,90] (n padres=161 y n profesores=84). Los resultados avalan la estabilidad temporal y su uso como medida de seguimiento o evolución, con un intervalo entre ambas aplicaciones de dos semanas.

II) Respecto a las evidencias de *validez* se analizó la estructura interna se analizó mediante un análisis factorial exploratorio (análisis de componentes principales) con rotación Promax con normalización Kaiser, obteniendo tres factores que explican el 91.82% de la varianza en la muestra de tipificación de padres y el 92.6% en la de profesores.

Cuando los informantes son los padres, los resultados de la prueba de esfericidad de Bartlett [$\chi 2$ (10) = 2629.521; p <0.000) y el índice Kaiser-Meyer-Olkin (KMO) de adecuación muestral de .764 mostraron la adecuación de los datos para la aplicación de un análisis factorial (ver Tabla S2).

Cuando los informantes son los padres, los resultados de la prueba de esfericidad de Bartlett [$\chi 2$ (10) = 2571.673; p <0.000) y el índice Kaiser-Meyer-Olkin (KMO) de adecuación muestral de .696 mostraron la adecuación de los datos para la aplicación de un análisis factorial (ver Tabla S2).

Tabla S2. Prueba de KMO y Bartlett (padres *versus* profesores)

		Padres	Profesores
Medida Kaiser-Meyer-Olkin de adecuación de muestreo		.764	.696
Prueba de esfericidad de Bartlett	Aprox. Chi-cuadrado	2629.521	2571.673
	gl	10	10
	Sig.	.000	.000

Fuente: BRIEF-P (adaptación española).

En la Tabla S3 se presentan las escalas clínicas que se adhieren a cada factor cuando los informantes son los padres, así, en saturan en: (i) Factor 1, Memoria de Trabajo y Planificación y Organización explicando el 61.98% de la varianza. (ii) Factor 2, Control Emocional e Inhibición explican el 16.38% de la varianza. (iii) Factor 3, configurado por la escala clínica Flexibilidad explica el 12.01% de la varianza.

Tabla S3. Matriz de componentes rotados y varianza explicada (informantes padres).

Padres			
Escalas Clínicas	Componentes		
	1	2	3
Memoria de Trabajo	.944		
Planificación y Organización	.940		
Control Emocional		940	
Inhibición		.884	
Flexibilidad			.993
Varianza explicada	61.983%	16.382%	12.014%

Método de extracción: análisis de componentes principales.
Método de rotación: Promax con normalización Kaiser.
Fuente: BRIEF-P (adaptación española).

En la Tabla S4 se presentan las escalas clínicas que se adhieren a cada factor cuando los informantes son los padres, así, en saturan en: (i) Factor 1, Planificación y Organización y Memoria de Trabajo explicando el 61.62 % de la varianza. (ii) Factor 2, Control Emocional e Inhibición explican el 16.38% de la varianza. (iii) Factor 3, configurado por la escala clínica Flexibilidad explica el 12.01% de la varianza.

Tabla S4. Matriz de componentes rotados y varianza explicada (informantes profesores).

Profesores			
Escalas Clínicas	Componentes		
	1	2	3
Planificación y Organización	.955		
Memoria de Trabajo	.950		
Control Emocional		.937	
Inhibición		.847	
Flexibilidad			.958
Varianza explicada	61.622%	16.777%	14.197%

Método de extracción: análisis de componentes principales.
Método de rotación: Promax con normalización Kaiser.
Fuente: BRIEF-P (adaptación española)."
