# Supplementary material for: Comparative Study of the Development of Executive Functions in Children: Transition from the First Cycle to the Second Cycle of Early Childhood Education
Source: Brain Sci. 2024 Dec 18;14(12):1273. doi: 10.3390/brainsci14121273 (PMC11674397; doi:10.3390/brainsci14121273)
Supplement: Supplementary file 1 [file brainsci-14-01273-s001.zip › PSYCHOMETRIC PROPERTIES IN SPANISH OF BRIEF_ENGLISH.pdf]

Supplementary Materials Psychometric Properties In Spanish Of BRIEF-P

Information extracted from:

Bausela-Herreras E, Luque-Cuenca T. Evaluación Conductual de la Función Ejecutiva-Versión Infantil (BRIEF-P, versión española): fiabilidad y validez. Acta de Investigación Psicológica. diciembre de 2017;7(3):2811-22.
DOI: 10.1016/j.aipprr.2017.11.002

"The results obtained from the study of the psychometric properties of the BRIEF-P in its Spanish adaptation are presented below:
I) Regarding reliability, **internal consistency** was considered: This was calculated using Cronbach's α coefficient, yielding the following results: (i) parents-males [α = .906], (ii) parents-females [α = .908], (iii) teachers-males [α = .901], and (iv) teachers-females [α = .903]. In all the student subsamples, the reliability coefficient obtained exceeds .90, which can be considered excellent. The reliability coefficients for the different scales and clinical indices of the BRIEF-P, according to the informant and sex, are presented below.

Table S1. Reliability statistics according to informants (parents versus teachers) and sex.

Informant	Sex	Cronbach's Alpha	Cronbach's Alpha Based on Standardized Items	Number of Items
Parents	Male	.906	.945	9
	Female	.908	.947	9
Teachers	Male	.901	.942	9
	Female	.903	.944	9

Source: BRIEF-P (Spanish adaptation).

b) Temporal consistency or test-retest stability: The Global Executive Function Index coefficients were [α=0.90] (n parents = 161, n teachers = 84). The results support temporal stability and its use as a measure for monitoring or evolution, with a two-week interval between applications.

II) Regarding evidence of validity, the internal structure was analyzed through an exploratory factor analysis (principal component analysis) with Promax rotation and Kaiser normalization, yielding three factors that explain 91.82% of the variance in the parent standardization sample and 92.6% in the teacher sample.

When the informants were the parents, the results of Bartlett's test of sphericity [χ^2 (10) = 2629.521; $p < 0.000$] and the Kaiser-Meyer-Olkin (KMO) measure of sampling adequacy (.764) demonstrated the suitability of the data for factor analysis (see Table S2).
When the informants were the teachers, the results of Bartlett's test of sphericity [χ^2 (10) = 2571.673; $p < 0.000$] and the Kaiser-Meyer-Olkin (KMO) measure of sampling

adequacy (.696) demonstrated the suitability of the data for factor analysis (see Table S2).

Table S2. KMO and Bartlett's test (parents versus teachers)

		Padres	Profesores
Kaiser-Meyer-Olkin Measure of Sampling Adequacy		.764	.696
Bartlett's Test of Sphericity	Aprox. Chi-cuadrado	2629.521	2571.673
	gl	10	10
	Sig.	.000	.000

Source: BRIEF-P (Spanish adaptation).

Table S3 presents the clinical scales that correspond to each factor when the informants are the parents. These saturate as follows: (i) Factor 1, Working Memory and Planning and Organization, explaining 61.98% of the variance. (ii) Factor 2, Emotional Control and Inhibition, explains 16.38% of the variance. (iii) Factor 3, consisting of the Flexibility clinical scale, explains 12.01% of the variance.

Table S3. Rotated component matrix and explained variance (parent informants).

Parents			
Clinical Scales	Components		
	1	2	3
Working Memory	.944		
Planning and Organization	.940		
Emotional Control		940	
Inhibition		.884	
Flexibility			.993
Explained Variance	61.983%	16.382%	12.014%

Extraction method: Principal Component Analysis.
Rotation method: Promax with Kaiser normalization.
Source: BRIEF-P (Spanish adaptation).

Table S4 presents the clinical scales that correspond to each factor when the informants are the parents. These saturate as follows: (i) Factor 1, Planning and Organization and Working Memory, explaining 61.62% of the variance. (ii) Factor 2, Emotional Control and Inhibition, explaining 16.38% of the variance. (iii) Factor 3, consisting of the Flexibility clinical scale, explains 12.01% of the variance.

Table S4. Rotated Component Matrix and Explained Variance (Teacher Informants).

Profesores			
Clinical Scales	Componentes		
	1	2	3
Planning and Organization	.955		
Working Memory	.950		
Emotional Control		.937	
Inhibition		.847	
Flexibility			.958
Explained Variance	61.622%	16.777%	14.197%

Extraction method: Principal Component Analysis.
Rotation method: Promax with Kaiser normalization.
Source: BRIEF-P (Spanish adaptation)."
